# Pillar[3]trianglamines: deeper cavity triangular macrocycles for selective hexene isomer separation†

**DOI:** 10.1039/d2sc00207h

**Published:** 2022-03-02

**Authors:** Yanjun Ding, Lukman O. Alimi, Jing Du, Bin Hua, Avishek Dey, Pei Yu, Niveen M. Khashab

**Affiliations:** Smart Hybrid Materials (SHMs) Laboratory, Advanced Membranes and Porous Materials Center, King Abdullah University of Science and Technology (KAUST) Thuwal 23955-6900 Kingdom of Saudi Arabia niveen.khashab@kaust.edu.sa; Key Laboratory of Polyoxometalate and Reticular Material Chemistry of Ministry of Education, Faculty of Chemistry, Northeast Normal University Changchun 130024 China

## Abstract

The separation of α-olefins and their corresponding isomers continues to be a big challenge for the chemical industry due to their overlapping physical properties and low relative volatility. Herein, pillar[3]trianglamine (P-TA) macrocycles were synthesized for the molecular-sieving-like separation of 1-hexene (1-He) selectively over its positional isomer *trans*-3-hexene (trans-3-He) in the vapor and liquid state. This allyl-functionalized macrocycle features a deeper cavity compared to the previously reported trianglamine host molecules. Solid–vapor sorption experiments verified the successful separation of 1-He from an equimolar mixture of 1-He and trans-3-He. Single-crystal structures and powder X-ray diffraction patterns suggest that this selective adsorption arises from the formation of a thermodynamically stable host–guest complex between 1-He and P-TA. A reversible transformation between the nonporous guest-free structure and the guest-containing structure shows that 1-He separation can be carried out over multiple cycles without any loss of performance. Significantly, P-TA can separate 1-He directly from a liquid isomeric mixture and thus P-TA modified silica sieves (SBA-15) showed the ability to selectively separate 1-He when utilized as a stationary phase in column chromatography. This capitalizes on the prospects of employing macrocyclic hosts as molecular recognition units in real-life separations for sustainable and energy-efficient industrial practices.

## Abstract 2

Crystalline allyl-functionalized trianglamine macrocycles (P-TA) that show a pillared-like cavity were successfuly prepared and employed for the robust molecular sieving of 1-He from vapor and liquid (in solution) isomeric mixtures.

## Graphical abstract

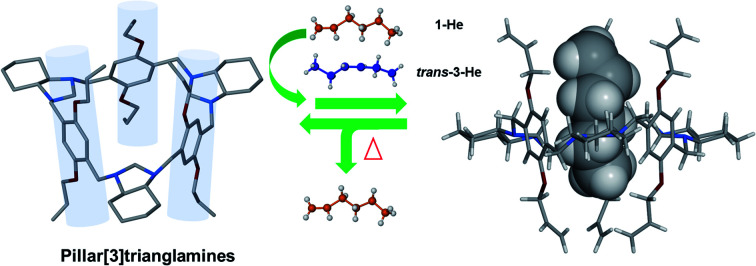

## Introduction

Higher α-olefins such as 1-hexene have a high industrial value as they are heavily used as comonomers in the production of commercial polymers like linear low density polyethylene (LLDPE).^1,2^ One of the major production methods of 1-He is the new ethylene recovery process developed by Lummus, which involves the separation of 1-He from 3-He.^3^ Such processes are plagued with poor selectivity due to alkene isomerization, alkyne semihydrogenation, chlorination/dehydrochlorination and other processes that result in regio- and stereoisomeric mixtures.^2^ These isomers are hard to separate by conventional methods due to their close boiling points and high volatility.^4–9^ Current practices depend on extraction and reactive extractive distillation, which are energy intensive and operationally complex.^3,10–12^ Furthermore, the separation of these olefins can be very problematic due to the tendency of their highly reactive double bonds to undergo thermally induced polymerizations at elevated temperatures.^13^ An interesting alternative is adsorptive separation using porous materials such as zeolites, metal–organic frameworks (MOFs) and organic porous materials.^14–26^ Molecular entities such as nonporous adaptive crystals (NACs) and organic cages have also shown great potential in the separation of hydrocarbons with good selectivity and enhanced stability and processability.^27–39^ NACs of pillararene-based macrocycles have been the most promising for the practical application of separation and storage of hydrocarbons.^27^ Ogoshi's pioneering report on the vapor uptake in the cavity of pillararenes sparked a major interest in utilizing these macrocycles for selective separation.^40^ Huang *et al.* achieved the sorting of various isomeric hydrocarbon mixtures using NACs based on pillar[5]arene macrocycles.^30–36^ Yang's group successfully reported new NACs based on pillararene derivatives such as the leaning pillar[6]arene and geminiarene that showed excellent separation capabilities.^27,41–43^ Moreover, other macrocycles including biphen[3]arene, tiara[5]arene, and hybrid[3]arene showed interesting selectivity towards isomeric mixtures such as halogenated hydrocarbons.^44–46^

**Fig. 1:**
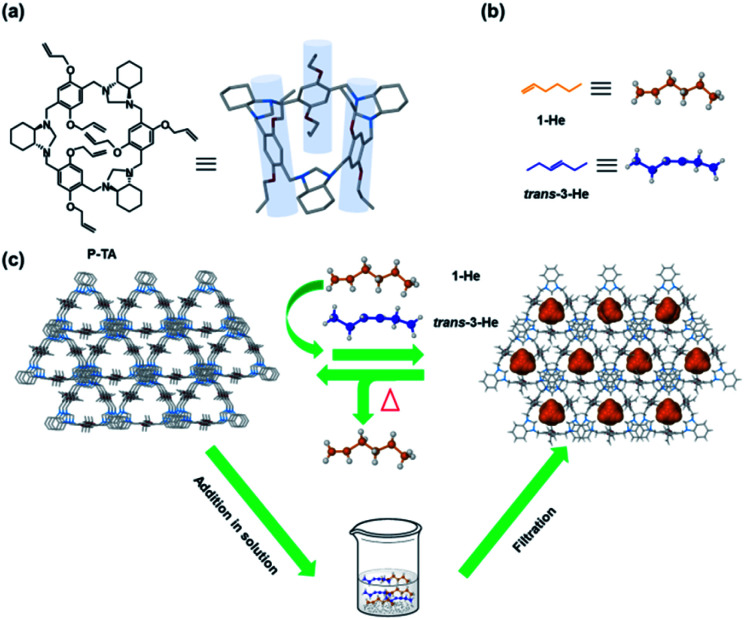
Chemical structures: (a) P-TA; (b) linear hexene isomers (1-He and trans-3-He). (c) Schematic representation of P-TA as a selective absorbent of 1-He from vapor and liquid isomeric mixtures.

Recently, we reported a series of trianglimine and trianglamine macrocycles for the adsorptive separation of hydrocarbons and haloalkanes.^47–49^ Attempts to employ our original system for linear hexene isomer separation were unsuccessful. A report by Huang and co-workers highlighted the impact of the macrocyclic cavity and hydrogen acceptors in the sorting of positional isomers using pillar[5]arenes.^32^ We thus ventured to design and prepare a new generation of triangular macrocycles with a deeper cavity for higher alkene separation. Herein, we present the synthesis of crystalline allyl-functionalized trianglamine macrocycles (P-TA) that showed a pillar-like cavity and can be employed for the robust molecular sieving of 1-He from vapor and liquid isomeric mixtures (Fig. 1). To better illustrate the utility of such host macrocycles for “on-demand” separations, soaking regular silica (SBA-15) in a solution of P-TA (in CH_2_Cl_2_) followed by slow evaporation, washing and drying provided P-TA modified SBA-15 that can be readily used in column chromatography (as a stationary phase) for 1-He separation. To the best of our knowledge, this is the first example of a selective adsorption of 1-He over trans-3-He employing a host–guest tailored molecular sieving technique in solution.

## Results and discussion

P-TA was synthesized starting from 2,5-dihydroxy-1,4-benzenedicarboxaldehyde (Fig. S1 and S2†) in 75% overall yield (Scheme S1†). The structure was characterized using NMR spectroscopy (Fig. S3 and S4†) and single crystal X-ray diffraction (SCXRD). SCXRD analysis showed a pillared chemical structure (Fig. 1a) and suggested that P-TA crystallizes in the trigonal crystal system, with a *P*3_1_ space group and the asymmetric unit containing one unit each of the macrocycle and dichloromethane (DCM) (Table S1 and Fig. S5†). They are packed in a head to tail fashion with interconnecting channels along the *c*-axis where DCM molecules occupied the channels (Fig. S6†). Thermogravimetric analysis (TGA) data revealed that P-TA loses DCM to become a guest-free or activated adsorptive material, which will be used in all further adsorption experiments (Fig. S7†). The powder X-ray diffraction (PXRD) patterns indicated that the activated P-TA was also crystalline even after activation at 120 °C under vacuum (Fig. S8†). The N_2_ gas sorption experiments showed that the activated P-TA was nonporous with a BET surface area of 3.9 m^2^ g^−1^ (Fig. S9†).

**Fig. 2:**
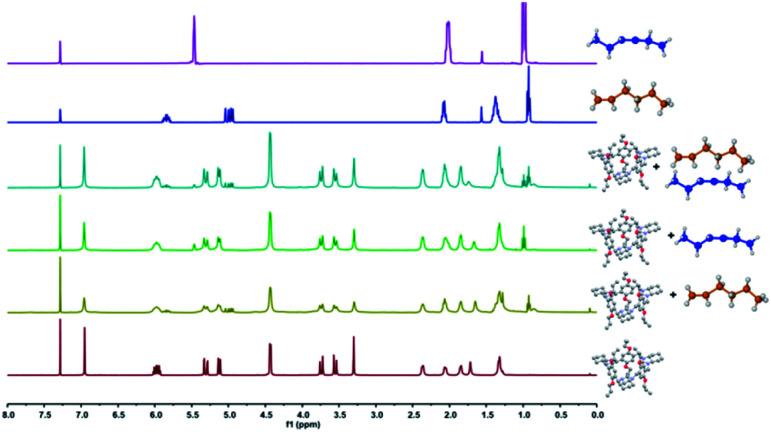
^1^H NMR spectra (400 MHz, chloroform-d, 298 K) of activated P-TA, activated P-TA after adsorption of vapor 1-He, activated P-TA after adsorption of vapor trans-3-He, activated P-TA after adsorption of the vapor 1-He/trans-3-He mixture for 16 h, 1-He and trans-3-He. The solid samples were dried after vapor adsorption and then dissovled in chloroform-d for analysis.

Solid–vapor sorption experiments were then conducted to investigate the selective uptake of activated P-TA towards 1-He, trans-3-He and their equimolar mixture at room temperature. The ^1^H NMR results showed the uptake of 1-He, trans-3-He and the selective adsorption of 1-He over trans-3-He with a high selectivity and an uptake ratio of *ca.* 4 : 1 (Fig. 2 and S10–S12†). PXRD patterns of activated P-TA displayed structural transformation after being exposed to 1-He and trans-3-He, suggesting their consequent adsorption.

Significantly, upon exposure to an equimolar mixture of 1-He and trans-3-He, the activated P-TA revealed a preferred adsorption of 1-He over trans-3-He (Fig. S13†). This supports that the pillared cavity can ultimately result in the selective adsorption of 1-He over trans-3-He. Attempting this separation with the original trianglamine (TA) resulted in no selectivity towards hexene isomer separation (Fig. S14†). Thermogravimetric analysis (TGA) data further confirmed the quantitative adsorption and the stable storage of 1-He and trans-3-He (Fig. S15 and S16†).

**Fig. 3:**
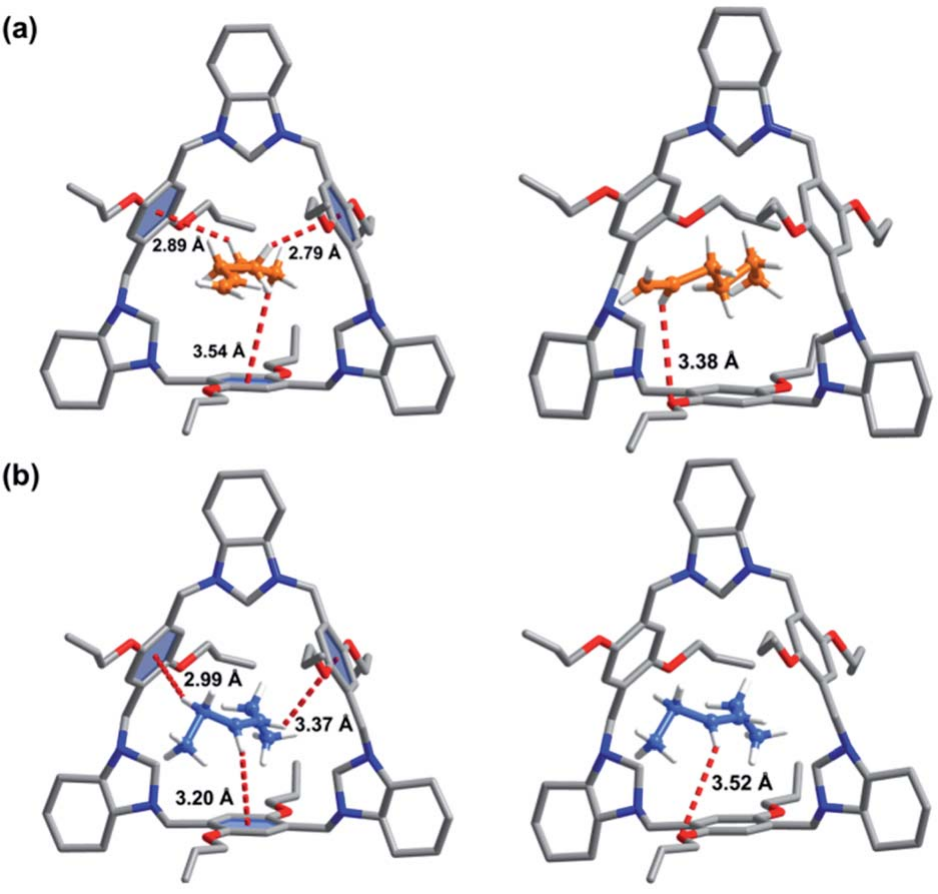
SCXRD of: (a) 1-He@P-TA; (b) *trans*-3-He@P-TA (The host–guest complex is stabilized by C–H⋯π and C–H⋯O interactions).

SCXRD patterns of P-TA loaded with 1-He or trans-3-He guest molecules were successfully obtained *via* crystallization in chloroform (ESI†). A 1 : 1 host–guest complex was formed for 1-He loaded P-TA (1-He@P-TA, Fig. 3a) and it crystallized in the non-centrosymmetric trigonal crystal system, with a chiral *R*3 space group (Table S2†). Each 1-He molecule is encapsulated in the intrinsic cavity of P-TA and stabilized by C–H⋯π and C–H⋯O interactions. The SQUEEZE program and the Olex2 function were further used to estimate the electron number in the cavity of P-TA.^50^ A solvent mask was used that calculated 53 electrons in a volume of 311 Å^3^ in the void per formula unit. This is consistent with the presence of one [C_6_H_12_] per formula unit. In the crystal structure of trans-3-He loaded P-TA (*trans*-3-He@P-TA, Fig. 3b), SCXRD analysis revealed that it crystallizes in the same trigonal crystal system and chiral space group (Table S2†). The asymmetric unit contains one unit of P-TA and half a unit of trans-3-He. The trans-3-He molecules sit inside the intrinsic cavity of P-TA, also stabilized by C–H⋯π and C–H⋯O interactions. The estimated electron number for three disordered trans-3-He molecules (27 electrons, 309 Å^3^ volume) was confirmed using PLATON SQUEEZE,^50^ which was consistent with the presence of 0.5 [C_6_H_12_] per formula unit. Notably, 1-He was calculated to have stronger noncovalent interactions with P-TA (C–H⋯π and C–H⋯O interactions) compared to trans-3-He (Fig. 3).

**Fig. 4:**
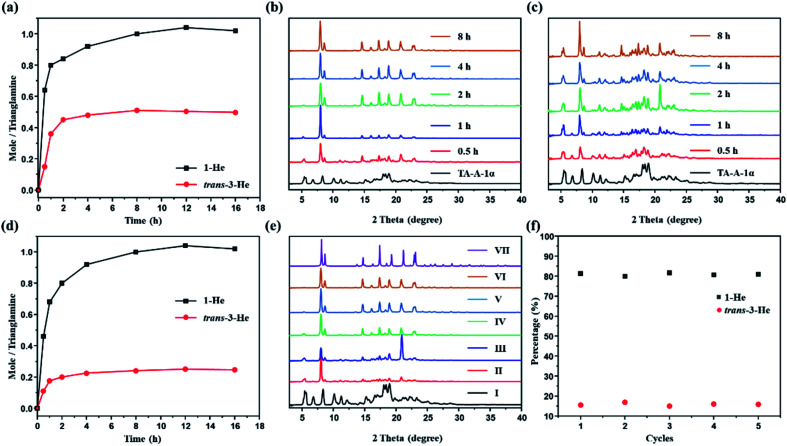
Time-dependent solid–vapor sorption plots. (a) Single component adsorption of activated P-TA upon exposure to 1-He and trans-3-He over time, respectively. Time-dependent PXRD patterns of activated P-TA upon exposure to (b) 1-He and (c) trans-3-He over time. (d) Adsorptive selectivity of 1-He and trans-3-He from their equimolar mixture. (e) Time-dependent PXRD patterns of activated P-TA upon exposure to an equimolar mixture of 1-He and trans-3-He (I) 0 h; (II) 0.5 h; (III) 1 h; (IV) 2 h; (V) 4 h; (VI) 8 h; (VII) simulated from the single crystal structure of 1-He@P-TA. (f) Relative uptake of 1-He and trans-3-He in activated P-TA for 16 h after activated P-TA is recycled 5 times.

Time-dependent solid–vapor sorption experiments were then performed on the activated P-TA upon exposure to 1-He, trans-3-He and their equimolar mixture. Fig. 4a shows that the uptake increases over time and reaches saturation at around 8 h (Fig. S17 and S18†). The uptake rates of 1-He and trans-3-He in the activated P-TA show a sharp increase in the initial 2 h. However, time-dependent studies clearly show that the uptake amount of 1-He was *ca.* 1.0 molecule per trianglamine at adsorption saturation, while only 0.5 molecule of trans-3-He was up taken. PXRD patterns of the activated P-TA gradually showed structural transformation after being exposed to 1-He and trans-3-He, triggered by guest adsorption (Fig. 4b and c). Interestingly, the uptake of 1-He increased over time and reached a saturation point (*ca.* 1.0 molecule per trianglamine) after 8 h. The uptake of trans-3-He was suppressed by competitive adsorption (*ca.* 0.2 molecule per trianglamine), implying a good adsorptive selectivity of 1-He (Fig. 4d and S19†). Gas chromatography (GC) showed that the ratio of 1-He adsorbed by the activated P-TA over the course of the experiment was over 84%, while trans-3-He only accounted for 16% (Fig. S20†). PXRD patterns of P-TA after the uptake of the 1-He/trans-3-He mixture vapor also changed with time. At the saturation point, it matched well with the pattern of 1-He@P-TA upon adsorption of 1-He (Fig. 4e). Time-dependent solid–vapor sorption experiments were also carried out using 1-He@P-TA with trans-3-He vapor as the competing guest. Over time, no structural transformation took place while the PXRD patterns of *trans*-3-He@P-TA gradually transformed to that of 1-He@P-TA after being exposed to 1-He vapor (Fig. S21†). These results further support that the complexation of P-TA with 1-He is thermodynamically more stable than that with trans-3-He.

One barrier in adsorption separation is the decreasing performance of adsorptive materials over time, because of their fouling or instability. For sustainable industrial practices, an adsorbent must be used reproducibly over multiple cycles. In this case, P-TA showed selective adsorption of 1-He over trans-3-He at least five times with no significant loss of efficiency (Fig. 4f). Moreover, the guest-loaded P-TA would directly transform back to the activated P-TA after the removal of guests *via* heating at 90 °C under vacuum, as supported by the PXRD experiments (Fig. S22†). The water stability of the activated P-TA was also tested and showed no structural degradation even after being emerged in an aqueous solution for 7 days as confirmed by PXRD (Fig. S23†).

**Fig. 5:**
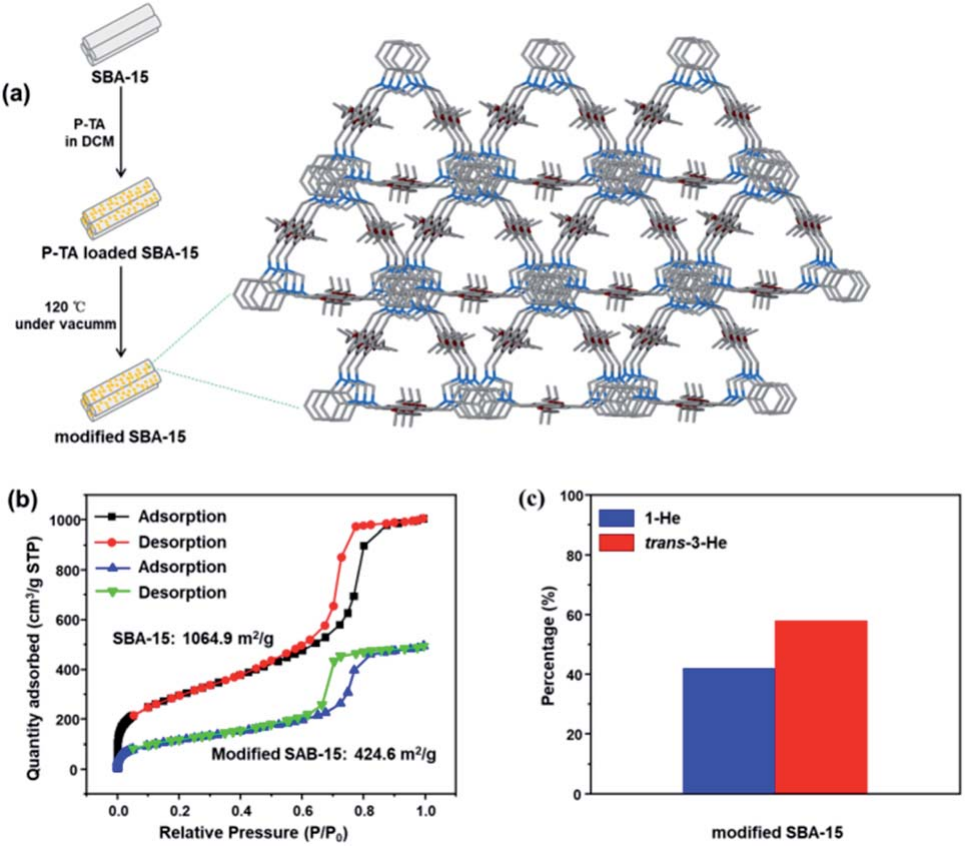
(a) Schematic illustration of P-TA modified SBA-15. (b) Nitrogen adsorption isotherm at 77 K of SBA-15 and modified SBA-15. (c) Relative amounts of 1-He and trans-3-He after the first run on column chromatography with P-TA modified SBA-15 as the stationary phase.

As these molecular entities have a relatively smaller surface area compared to extended frameworks, the kinetics of adsorption was significantly slower. Consequently, we tested our molecular motifs for solid–liquid separation of He isomers directly from solution as they are stable and not soluble in the presence of 1-He (Fig. S24†). Soaking P-TA crystals in a solution of 1-He/trans-3-He showed a selective uptake of 1-He over trans-3-He (Fig. S25†). Moreover, to improve on the practical application of these molecular recognition units, column chromatography grade silica (pore sizes: 7.5 nm), SBA-15, was modified by crystallization of P-TA in the mesopores.^51^ The modified SBA-15 was washed three times to remove any P-TA crystallization on the surface while maintaining the micro-crystals in the mesopores (Fig. 5a and S26, S27†). The main driving force for micro-crystal formation in the pores is physical adsorption, which is similar to capillary action. As the apparent surface area of SBA-15 remains constant, the loading of nonporous P-TA decreased the overall surface area. The BET surface area and pore volume (at *P*/*P*_0_ = 0.99) decreased from 1064.9 m^2^ g^−1^ to 424.6 m^2^ g^−1^ and 1.55 cm^3^ g^−1^ to 0.76 cm^3^ g^−1^, respectively (Fig. 5b and S28†). Employing P-TA modified SBA-15 as a stationary phase in column chromatography readily increased the amount of trans-3-He after 1 run (60% trans-3-He and 40% 1-He) suggesting a proper differentiation between the two isomers and the capture of 1-He as the preferred guest (Fig. 5c and S29a†). As a comparison, unmodified SBA-15 could not distinguish 1-He and *trans*-3-He showing no separation (Fig. S29b†).

## Conclusions

In summary, we have investigated the adsorptive properties of the pillared allyl-functionalized trianglamine (P-TA) towards the selective separation of linear hexene isomers. The activated P-TA could selectively capture 1-He over trans-3-He with over 84% selectivity. The formation of a host–guest complex between 1-He and P-TA is thermodynamically more stable. Compared to the parent TA, the introduction of allyl group in P-TA expanded its space and reshaped the cavity to selectively host longer chain hydrocarbons. Although molecular adsorbents such as P-TA show slow kinetics in solid–vapor sorption experiments, their stability and recyclability make it possible for them to be used directly as solid–liquid adsorbents. Most importantly, these host macrocycles can be used as smart recognition units to improve the molecular sieving of SBA-15. The stability, recyclability and ease of fabrication and tuning of P-TA make this class of macrocycles the ideal hosts for molecular-sieving-like separations of a wide range of industrially valuable isomeric compounds.

## Data availability

Characterization data including NMR, TGA and crystal data are included in the ESI file.†

## Author contributions

Y. D. performed the major experiments. L. A., B. H., A. D., and P. Y. contributed to the characterization experiments. J. D. helped with the computational calculations.N. M. K. supervised the work and finalized the paper.

## Conflicts of interest

There are no conflicts to declare.

## Supplementary Material

SC-013-D2SC00207H-s001

SC-013-D2SC00207H-s002
