# Supplementary material for: Pillar[3]trianglamines: deeper cavity triangular macrocycles for selective hexene isomer separation
Source: Chem Sci. 2022 Mar 2;13(11):3244–8. doi: 10.1039/d2sc00207h (PMC8926253; doi:10.1039/d2sc00207h)
Supplement: SC-013-D2SC00207H-s001 [file SC-013-D2SC00207H-s001.pdf]

Electronic Supplementary Material (ESI) for Chemical Science.
This journal is © The Royal Society of Chemistry 2022

Electronic Supplementary Information

Pillar[3]trianglamines: Deeper Cavity Triangular Macrocycles for Selective Hexene Isomers Separation

Yanjun Ding,[1] Lukman O. Alimi,[1] Jing Du,[2] Bin Hua,[1] Avishek Dey,[1] Pei Yu[1] and Niveen M. Khashab[1]*

[1] Smart Hybrid Materials (SHMs) Laboratory, Advanced Membranes and Porous Materials Center, King Abdullah University of Science and Technology (KAUST), Thuwal 23955-6900, Kingdom of Saudi Arabia.

[2]Key Laboratory of Polyoxometalate and Reticular Material Chemistry of Ministry of Education, Faculty of Chemistry, Northeast Normal University, Changchun, 130024, China.

1. Experimental Procedures

1.1. Materials. All chemicals were purchased from commercial sources and used as received.

1.2. Synthesis of trianglamine TA. Trianglamine **TA** was prepared according to previous report.[1] ^{1}H NMR (400 MHz, Chloroform-d) δ 7.15 (s, 2H), 4.07 (d, *J* = 14.0 Hz, 1H), 3.28 (s, 1H), 3.16 (d, *J* = 14.0 Hz, 1H), 2.30 – 2.28 (m, 1H), 2.04 – 2.02 (m, 1H), 1.84 – 1.82 (m, 1H), 1.33 – 1.27 (m, 2H); ^{13}C NMR (101 MHz, Chloroform-d) δ 138.04, 127.58, 69.13, 57.68, 29.44, 24.59. HRMS (ESI) calcd for $C_{45}H_{61}N_6$ [$(M+H)^+$]: 685.4958, Found: 685.5024.

1.3. Synthesis of ligand Da-A. A solution of **Da** (498.4 mg, 3.0 mmol) and allyl bromide (907.4 mg, 7.5 mmol) in acetonitrile (50 mL) was heated at reflux with powdered K_2CO_3 (1.66 g, 12 mmol) for 4 h. The solvent was evaporated and the residue was dissolved in CH_2Cl_2 and extracted with aqueous Na_2CO_3 (5%). The organic phase was dried over $NaSO_4$, evaporated and dried in vacuo to get the crude product in 83% yield, which was directly used in the next step. ^{1}H NMR (400 MHz, Chloroform-d) δ 10.53 (s, 2H), 7.44 (s, 2H), 6.06 (ddt, J = 17.2, 10.5, 5.2 Hz, 2H), 5.44 (dq, J = 17.2, 1.5 Hz, 2H), 5.34 (dq, J = 10.6, 1.4 Hz, 2H), 4.67 (dt, J = 5.2, 1.6 Hz, 4H); ^{13}C NMR (101 MHz, Chloroform-d) δ 189.28, 154.90, 132.22, 129.51, 118.62, 112.29, 69.93.

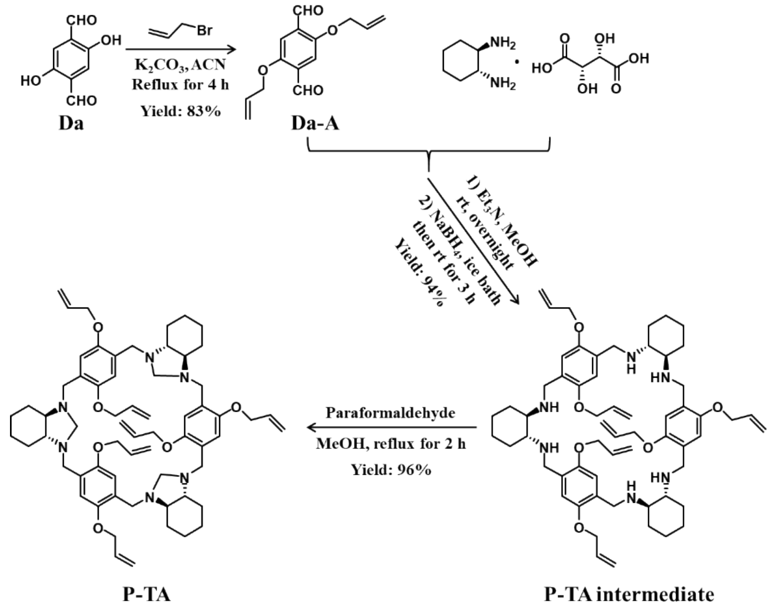

Scheme S1. Synthetic scheme of pillar[3]trianglamine **P-TA**.

1.4. Synthesis of P-TA intermediate. A mixture of (R, R)-(+)-1,2-Diaminocyclohexane L-Tartrate (528.6 mg, 2.0 mmol), **Da-A** (492.5 mg, 2.0 mmol), MeOH (20 mL) and triethylamine (0.7 mL) were stirred at room temperature overnight. The mixture was cooled in an ice bath and sodium borohydride (228 mg, 6 mmol) was added over one hour. After the system had been stirred for a further three hours at room temperature, the solvents were removed in vacuo and the residue was extracted with dichloromethane and aqueous sodium carbonate (5%). The organic solution was dried over $NaSO_4$, evaporated and dried in vacuo. Trianglamine **P-TA intermediate** was obtained in a yield of 94%. HRMS (ESI) calcd for $C_{60}H_{85}N_6O_6$ [$(M+H)^+$]: 985.6531, Found: 985.6526.

1.5. Synthesis of Pillared Trianglamine P-TA. **P-TA intermediate** (492.7 mg, 0.5 mmol) and paraformaldehyde (180 mg, 6 mmol) in CH_3OH (10 mL) was stirred at 70 °C for 2 h. The solvent was evaporated and the residue was dissolved in CH_2Cl_2 and extracted with aqueous Na_2CO_3 (5%). The organic phase was dried over $NaSO_4$, evaporated and dried in vacuo to get the crude product in 96% yield. Crude product was purified via crystallization from dichloromethane. ^{1}H NMR (400 MHz, Chloroform-d) δ 6.93 (s, 6H), 5.95 (ddt, J = 17.3, 10.4, 5.1 Hz, 6H), 5.28 (dq, J = 17.3, 1.7 Hz, 6H), 5.10 (dq, J = 10.6, 1.6 Hz, 6H), 4.42 (d, J = 1.6 Hz, 12H), 3.71 (d, J = 14.7 Hz, 6H), 3.53 (d, J = 14.6 Hz, 6H), 3.28 (s, 6H), 2.35 – 2.33 (m, 6H), 2.04 – 2.02 (m, 6H), 1.83 – 1.81 (m, 6H), 1.31 – 1.25 (m, 12H); ^{13}C NMR (101 MHz, Chloroform-d) δ 150.12, 133.94, 127.09, 117.01, 113.23, 77.94, 69.60, 69.30, 51.22, 29.63, 24.66. HRMS (ESI) calcd for $C_{63}H_{85}N_6O_6$ [$(M+H)^+$]: 1021.6531, Found: 1021.6722.

1.6. Single Crystal Growth. Single crystals of the pillared trianglamine **P-TA** were grown by slow evaporation of dichloromethane solution at room temperature. Single crystals of **1-He@P-TA** were obtained as follows: firstly mixed **P-TA** with 1-He, then sonicated it for 10 min and filtered the mixture through a PTFE membrane (220 nm) to get a clear solution, finally colorless single crystals appeared by slow evaporation of 1-He over several days. Single crystals of ***trans*-3-He@P-TA** were grown via a vapor diffusion

method: **P-TA** dissolved in $CHCl_3$ in a small vial was placed in a large vial containing *trans*-3-He to allow *trans*-3-He diffusion into the $CHCl_3$ solution.

1.7. Adsorption Material Activation. The desolvated **P-TA** crystals were prepared under vacuum at 120 ℃ overnight. The activated adsorptive separation materials (activated **P-TA**) after adsorption could be regenerated to release the adsorbed guests upon heating at 90 ℃ under vacuum overnight, and the released guests could be collected via a condensation setup.

1.8. Adsorption Experiments for linear hexene isomers vapor. An open 5 mL vial containing 10 mg of adsorbent was placed in a sealed 20 mL vial containing 1 mL of solvents (1-He, *trans*-3-He or an equimolar mixture of 1-He and *trans*-3-He) at room temperature. The uptake capacity of adsorbents was measured at different time intervals by completely dissolving the samples in $CDCl_3$ and measuring the ratio of 1-He or *trans*-3-He by ^1H NMR, respectively. Uptake capacity values were determined from the ratio of each isomer peaks using ^1H NMR and GC following literature protocols.[2]

2. Methods

2.1. Solution NMR. NMR spectra were recorded on Bruker-400 (400 MHz for ^1H; 101 MHz for ^{13}C) instruments internally referenced to $SiMe_4$ signal.

2.2. Thermogravimetric Analysis. Thermogravimetric analysis (TGA) was carried out using a TGA Q50 analyzer (TA Instruments) with an automated vertical overhead thermobalance. The samples were heated at 10 °C/min from 25 to 800 °C using N_2 as the protective gas.

2.3. Nitrogen Adsorption Experiment. Low-pressure gas adsorption measurement was performed on a Micromeritics Accelerated Surface Area and Porosimetry System (ASAP) 2020 surface area analyzer. Samples were degassed under dynamic vacuum for 12 h at 60 °C prior to each measurement. N_2 isotherms were measured using a liquid nitrogen bath (77 K).

2.4. Powder X-Ray Diffraction. Powder X-ray diffraction (PXRD) patterns were obtained using a D8 ADVANCE Twin X-ray diffractometer (40 KV, 40 mA) with the Cu Kα radiation (λ = 1.54178 Å). Data were measured over the range of 3−40° in 2°/min steps.

2.5. Single Crystal X-ray Diffraction. Single crystal X-ray diffraction data were recorded on a Bruker D8 Venture equipped with a digital camera diffractometer using graphite-monochromated Cu Kα (λ= 1.54178 Å) or Mo Kα (λ= 1.54178 Å) radiation for the crystal structures. Raw data were integrated using Bruker AXS SAINT software and absorption corrections were applied using SADABS.[3,4] All structures were solved with the ShelXT structure solution program using Intrinsic Phasing and refined with the ShelXL refinement package using Least Squares minimization operated in the OLEX2 interface.[5] All non-hydrogen atoms were refined anisotropically. In the final refinement, a twin law (TWIN -1 -1 0 0 1 0 0 0 -1 2; BASF = 0.12) was required for the structural refinement of **1-He@P-TA**. Based on theanalysis of ^1H NMR and TGA as well as the solvent mask calculation results, the asymmetric unit contains one unit of **P-TA** and one unit of 1-He in the **1-He@P-TA**; While *trans*-3-He molecules sit inside the intrinsic cavity of **P-TA** with 0.5 occupancy in the ***trans*-3-He@P-TA**. The hydrogen atoms on organic carbon atoms were fixed in calculated positions. Crystal data and structural refinement for **1-He@P-TA** and ***trans*-3-He@P-TA** are listed in Table S2.

2.6. Gas Chromatography. Gas Chromatographic (GC) Analysis: GC measurements were carried out using a J&W (122-1364) instrument configured with an FID detector and a DB-624 column (60 m × 0.25 mm × 1.4 μm). The following GC method was used: the oven was programmed from 40 °C ramped in 10 °C/min increments to 240 °C with 26 min hold. The total run time was 50 min and the injection temperature was 250 °C. The detector temperature was 260 °C with hydrogen, air, and make-up flow rates of 35, 350, and 30 mL/min, respectively. The helium (carrier gas) flow rate was 3.0 mL/min. The samples were injected in the splitless mode.

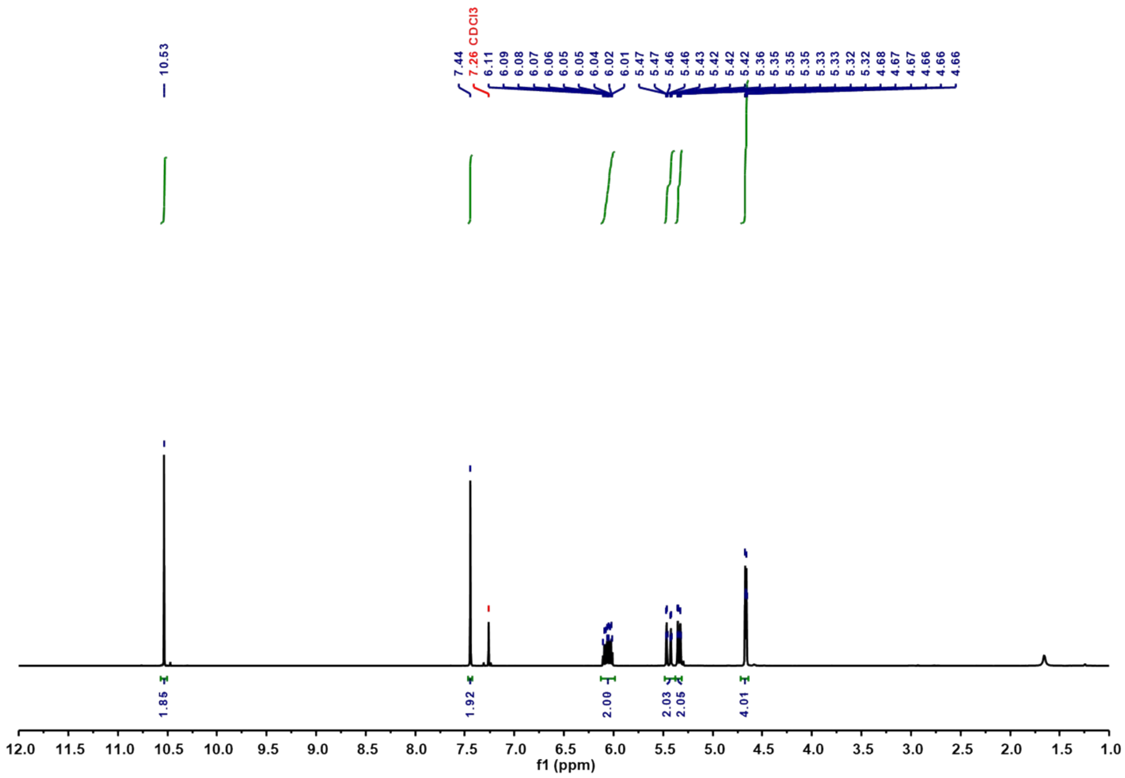

Figure S1. ^{1}H NMR spectrum (400 MHz, 298K, CDCl3) of ligand **Da-A**.

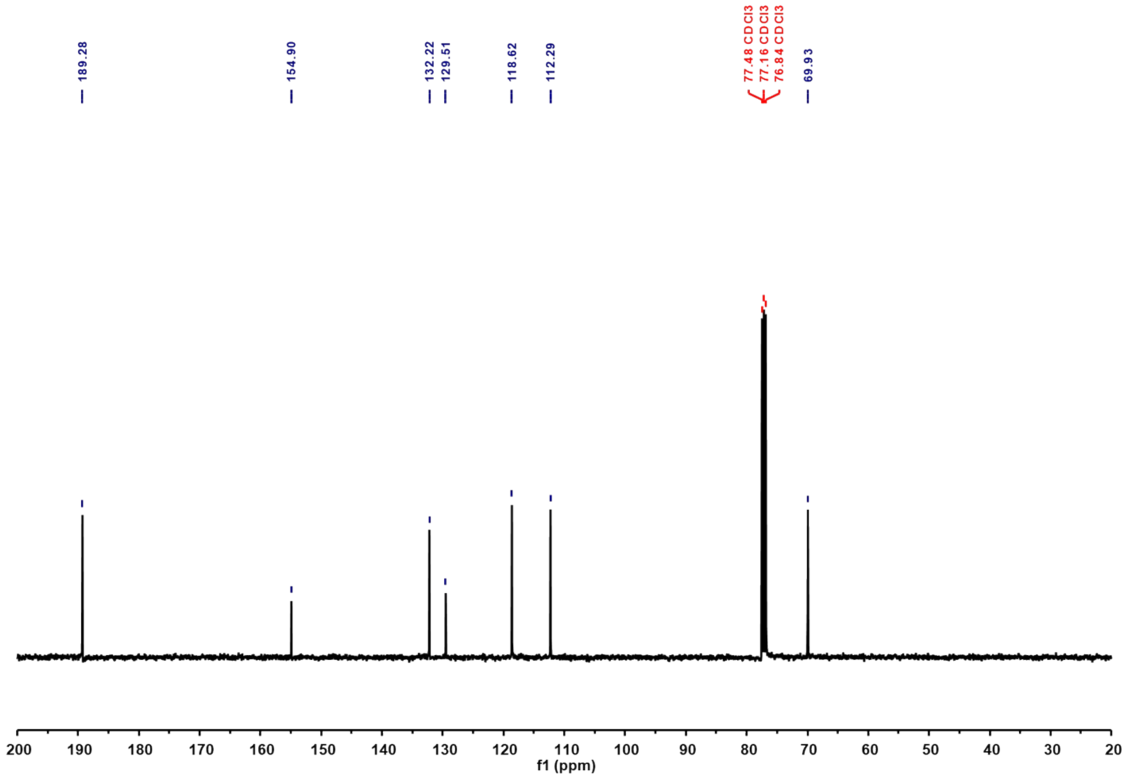

Figure S2. ^{13}C NMR spectrum (101 MHz, 298K, $CDCl_3$) of ligand **Da-A**.

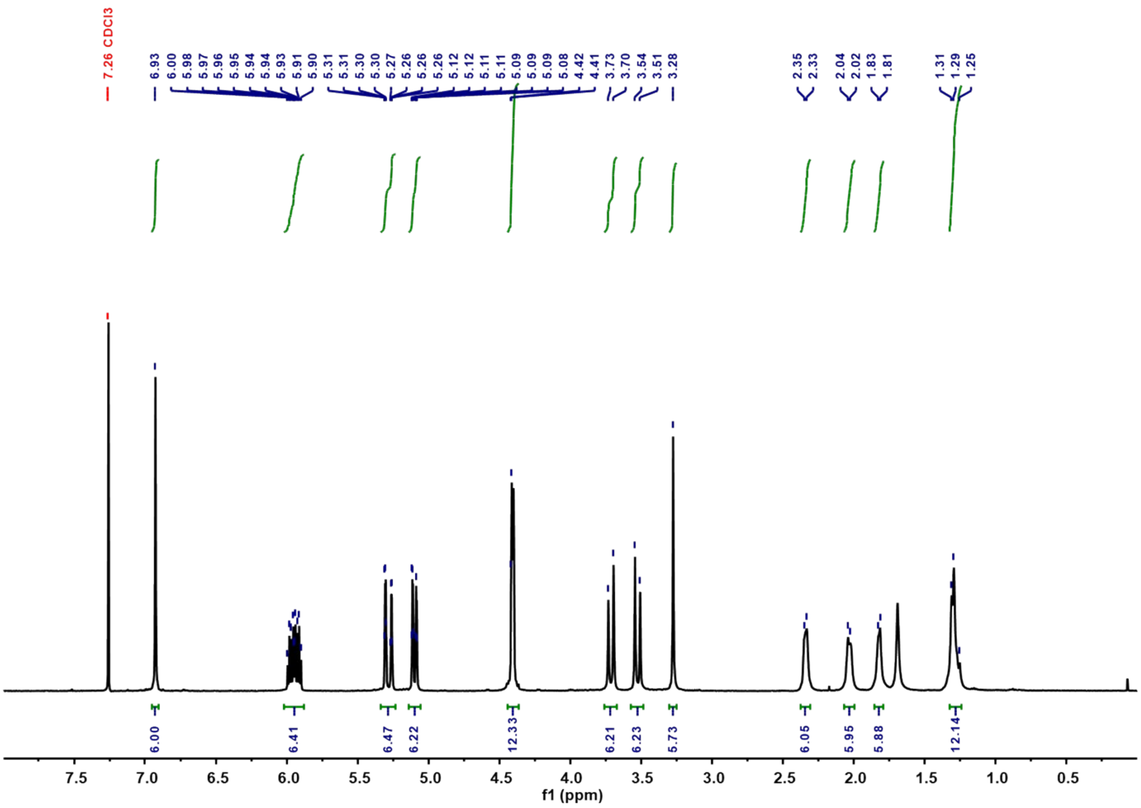

Figure S3. ^{1}H NMR spectrum (400 MHz, 298K, $CDCl_3$) of pillar[3]trianglamine **P-TA**.

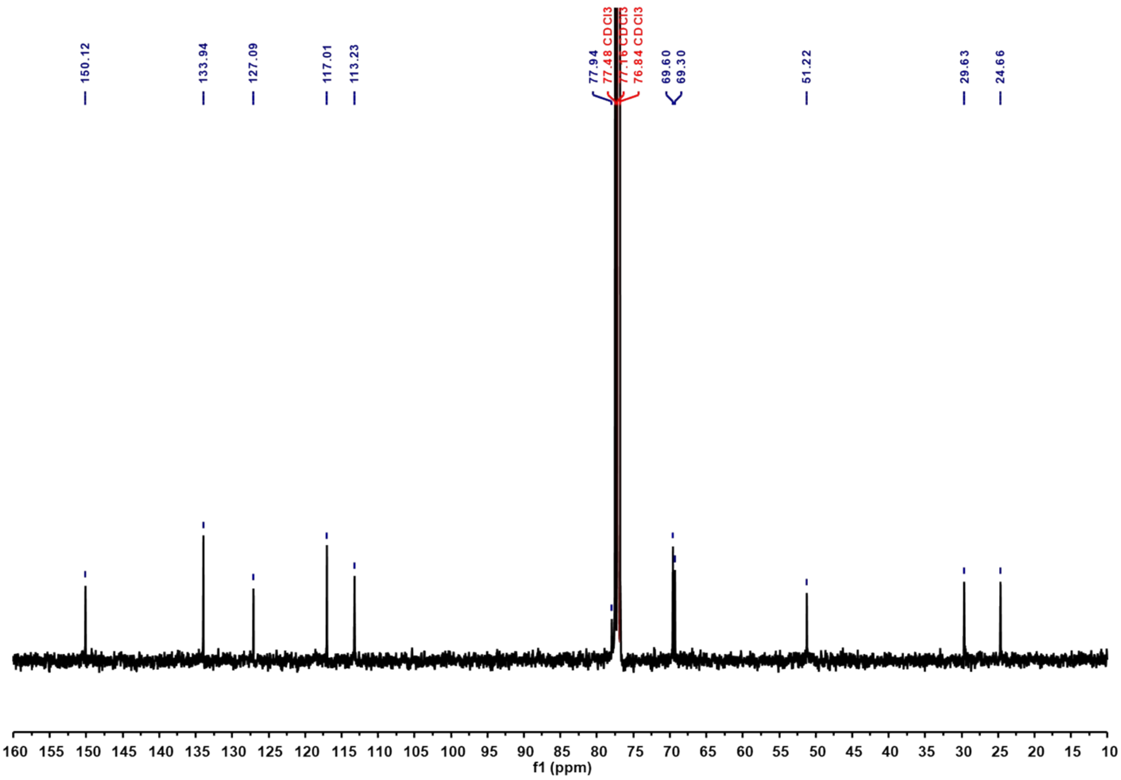

Figure S4. ^{13}C NMR spectrum (101 MHz, 298K, $CDCl_3$) of pillar[3]trianglamine **P-TA**.

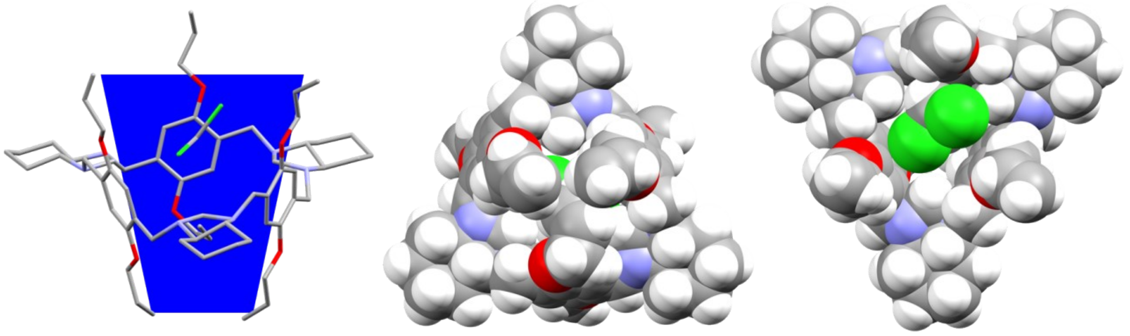

Figure S5. The asymmetric unit and the space filling structure of crystalline **P-TA**.

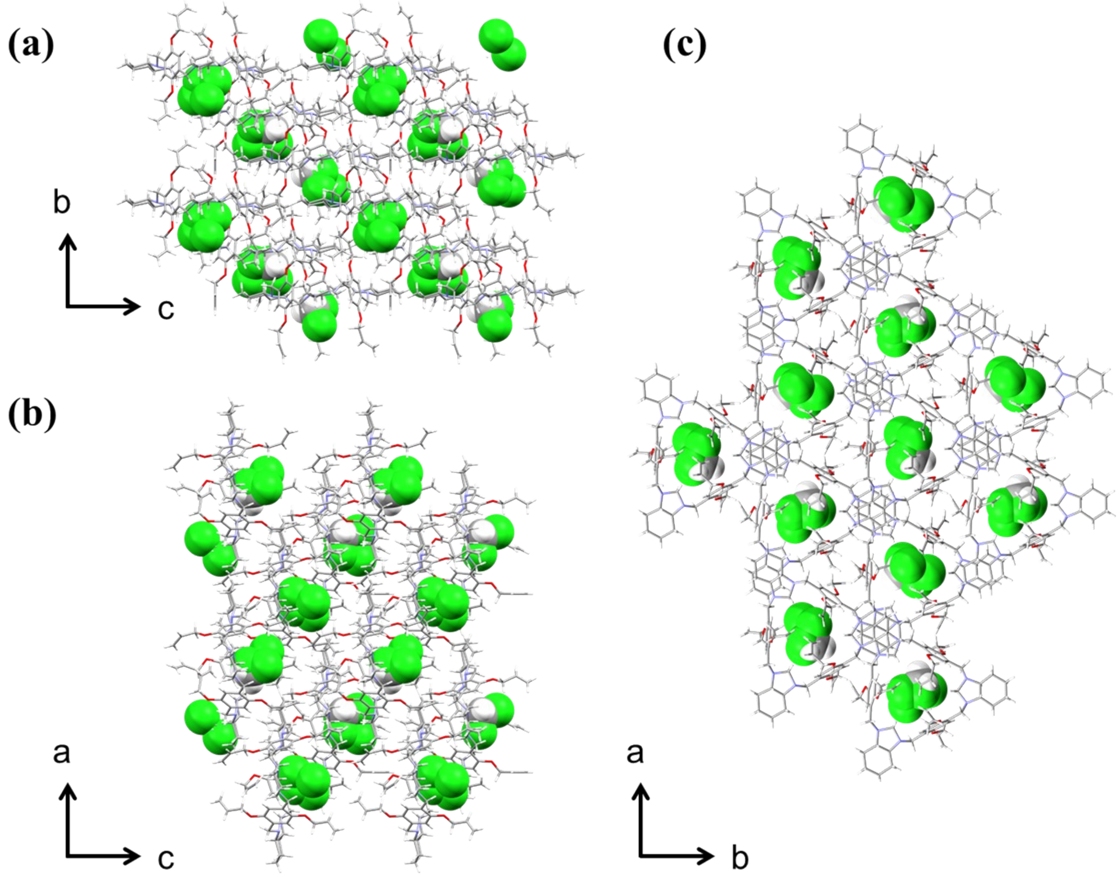

Figure S6. Packing arrangement of crystalline **P-TA** along (a) *a*-axis (b) *b*-axis and (c) *c*-axis.

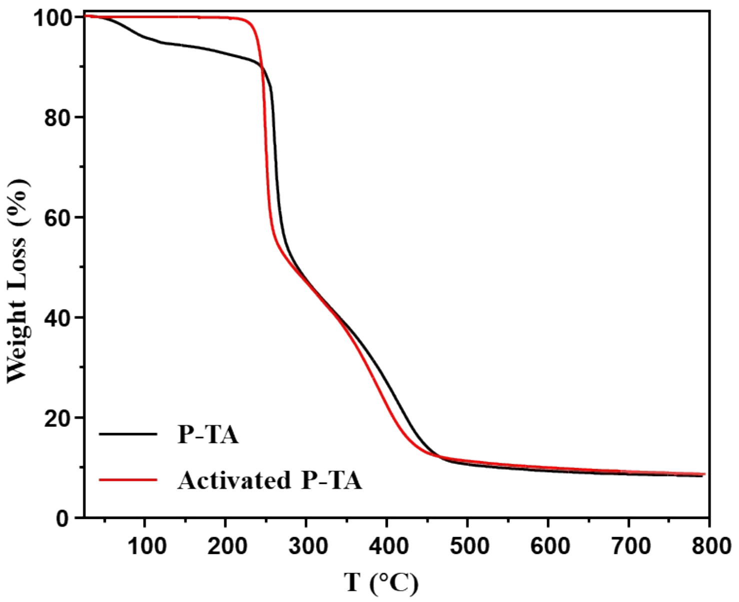

Figure S7. Thermogravimetric analysis: the as synthesized crystalline **P-TA** and activated **P-TA**.

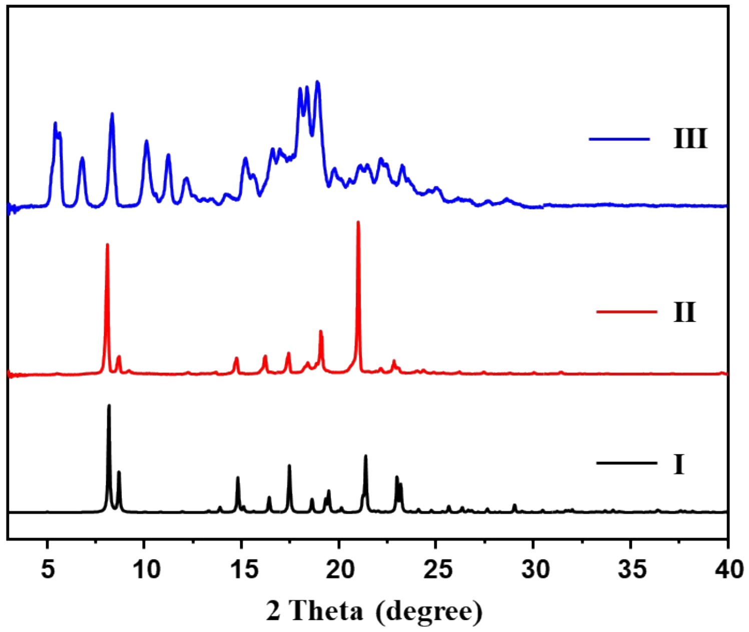

Figure S8. The PXRD patterns: (I) simulated from the single crystal structure of **P-TA**; (II) experimental from **P-TA** crystals; (III) activated **P-TA**.

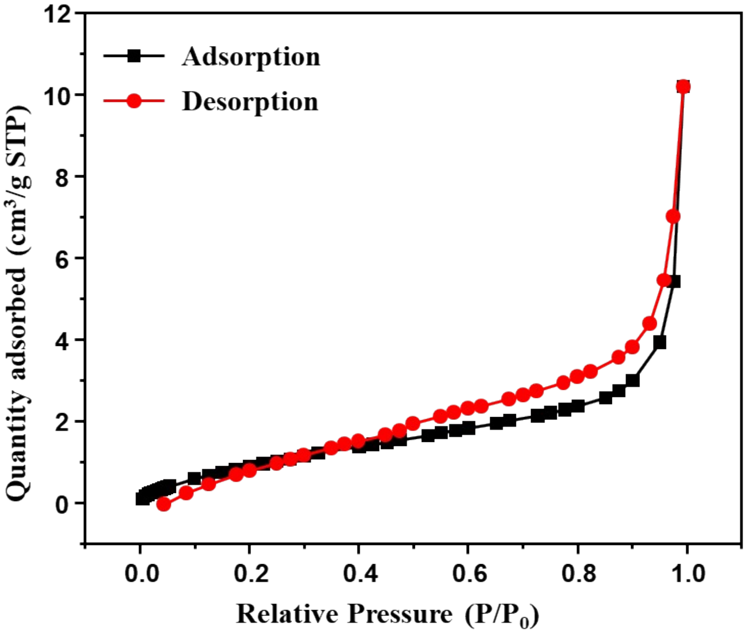

Figure S9. Nitrogen adsorption isotherm at 77 K for activated **P-TA**. The calculated BET surface area is 3.9 m^2/g.

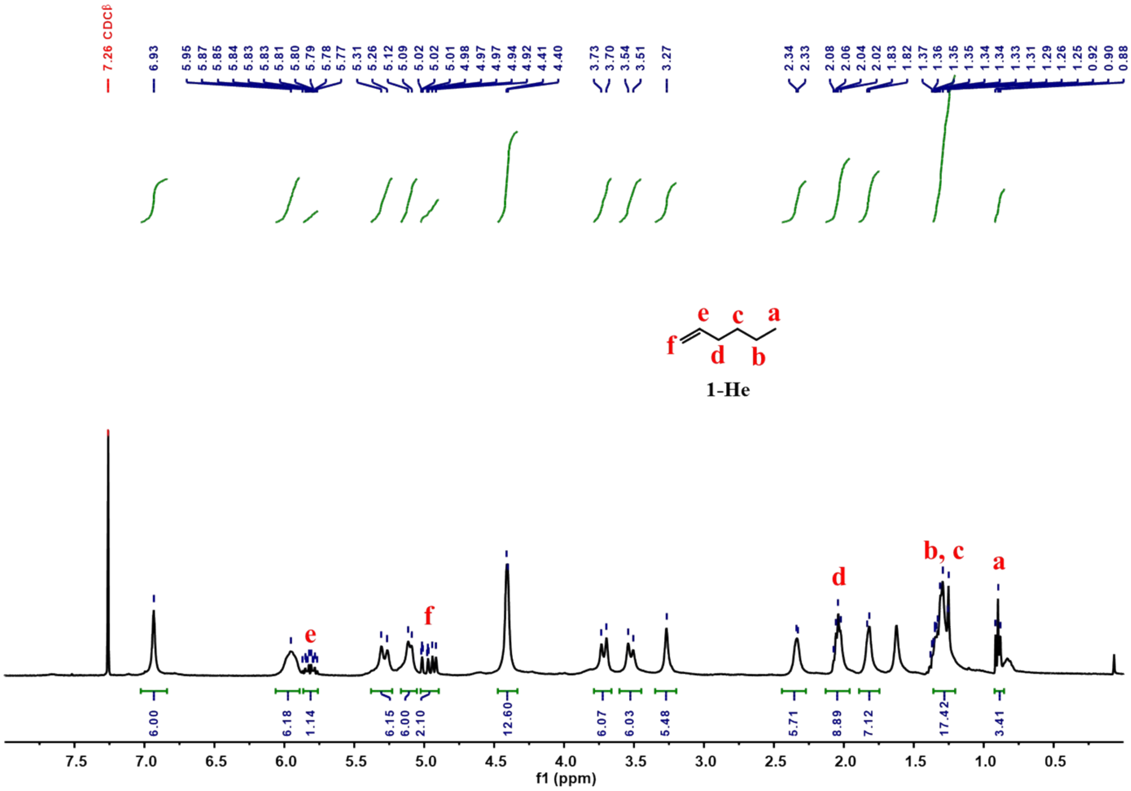

Figure S10. ^{1}H NMR spectra (400 MHz, chloroform-d, 298 K) of activated **P-TA** after adsorption of 1-He for 16 h.

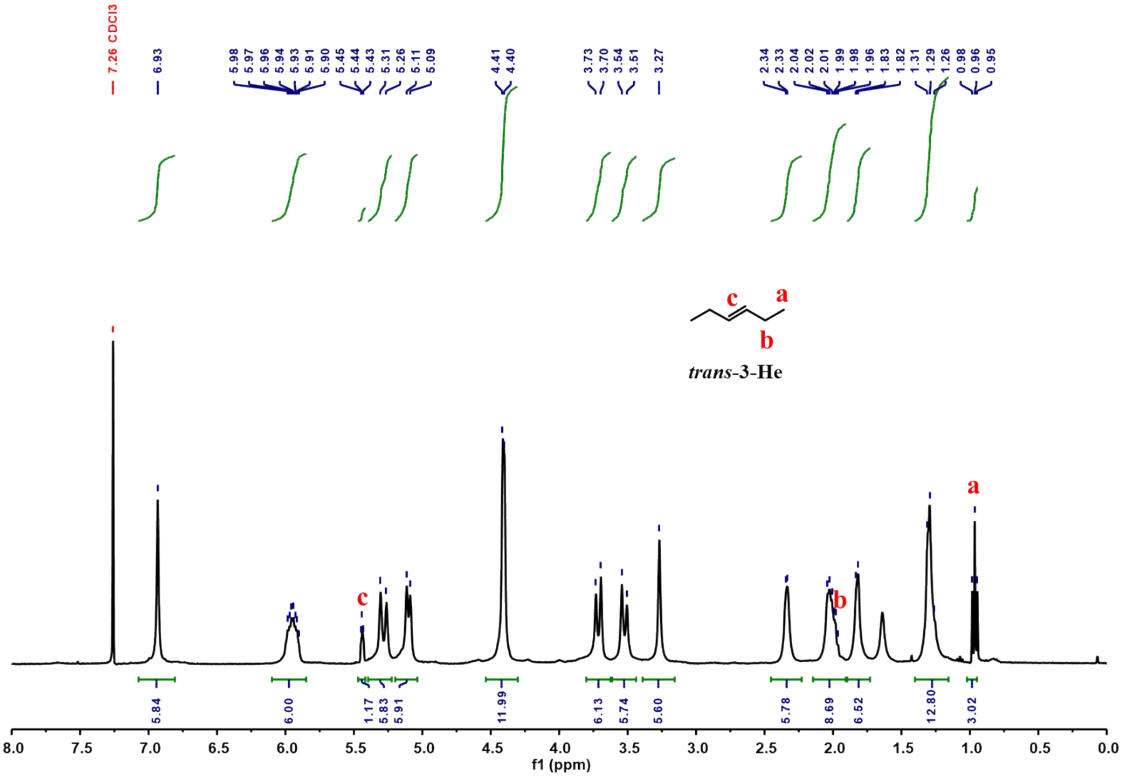

Figure S11. ^{1}H NMR spectra (400 MHz, chloroform-d, 298 K) of activated **P-TA** after adsorption of *trans*-3-He for 16 h.

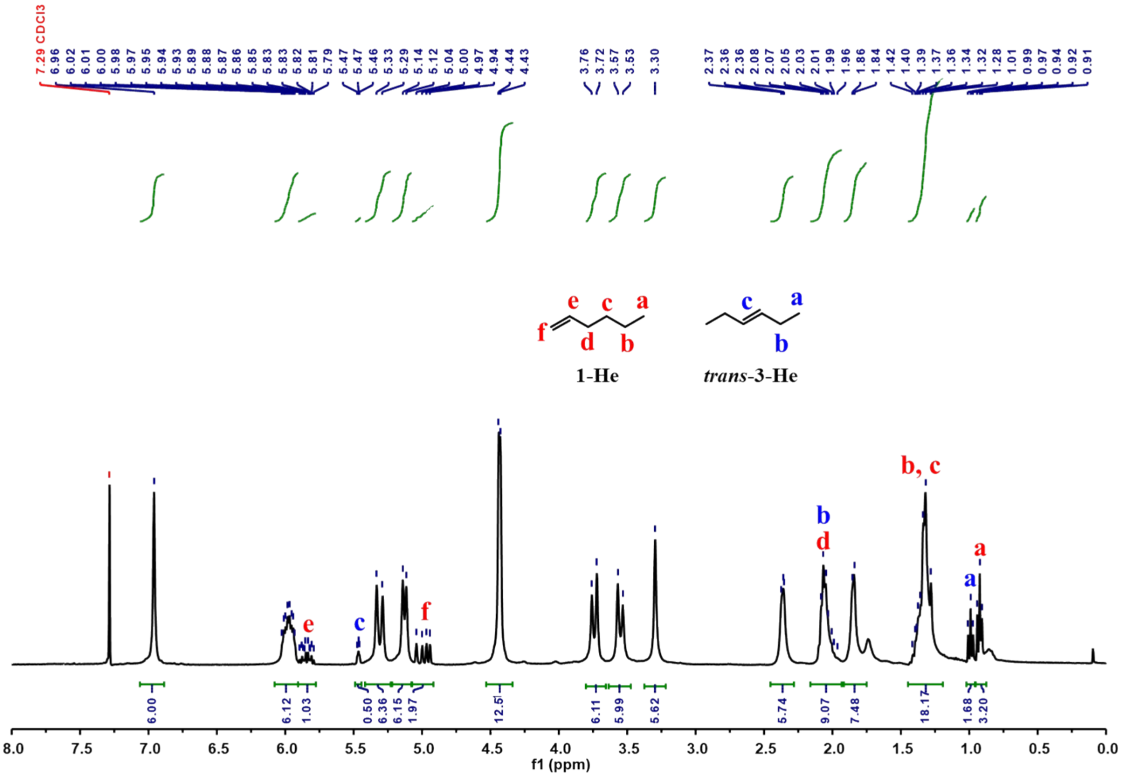

Figure S12. ^{1}H NMR spectra (400 MHz, chloroform-d, 298 K) of activated **P-TA** after adsorption of 1-He/*trans*-3-He mixtures for 16 h.

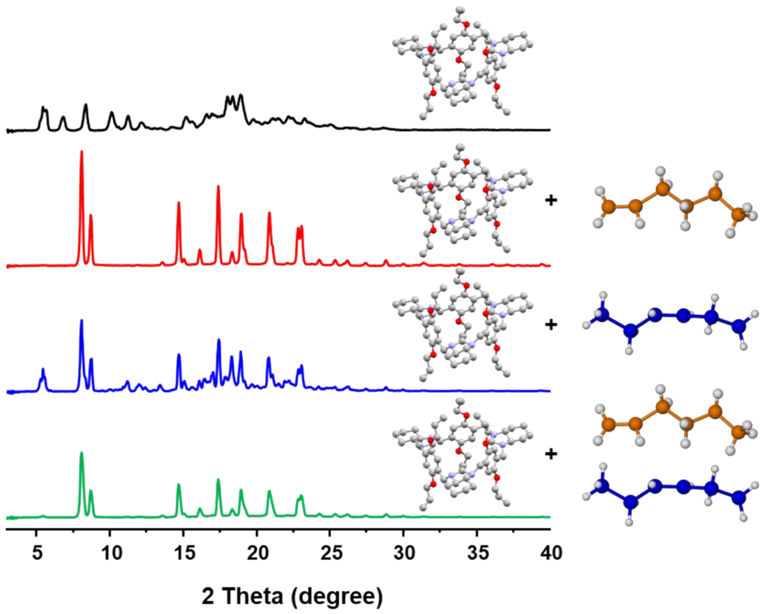

Figure S13. Experimental PXRD patterns of activated **P-TA** (black); activated **P-TA** after being exposed to 1-He (red); activated **P-TA** after being exposed to *trans*-3-He (blue); activated **P-TA** after being exposed to 1-He/*trans*-3-He mixtures for 16 h (green).

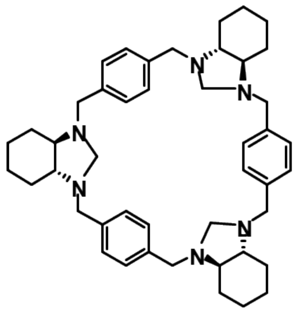

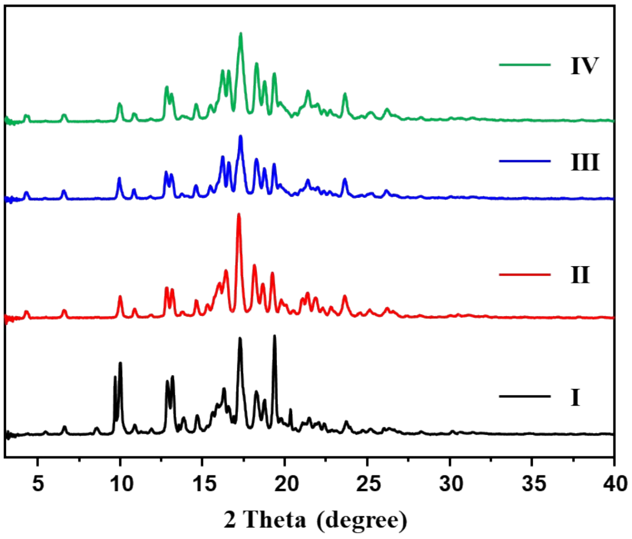

Figure S14. Chemical structure of **TA** and experimental PXRD patterns of (I) activated **TA**; (II) activated **TA** after being exposed to 1-He; (III) activated **TA** after being exposed to *trans*-3-He; (IV) activated **TA** after being exposed to 1-He/*trans*-3-He mixtures for 16h.

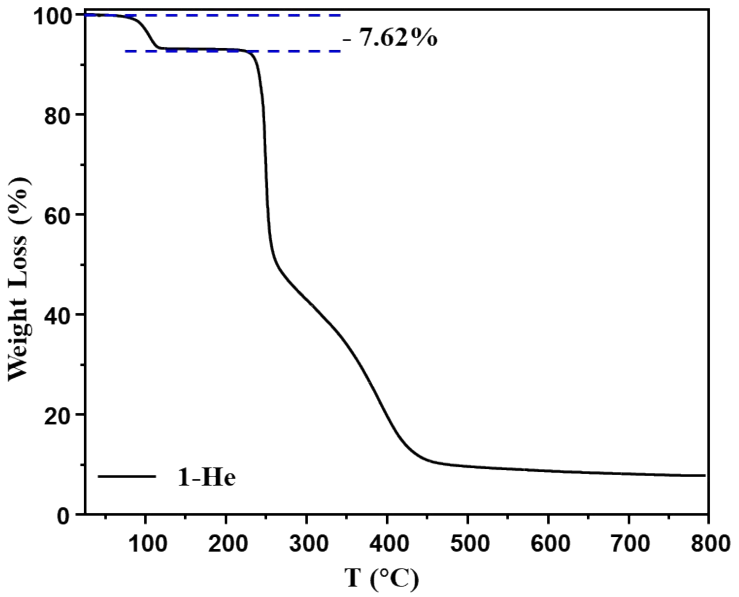

Figure S15. Thermogravimetric analysis of activated **P-TA** after adsorption of 1-He.

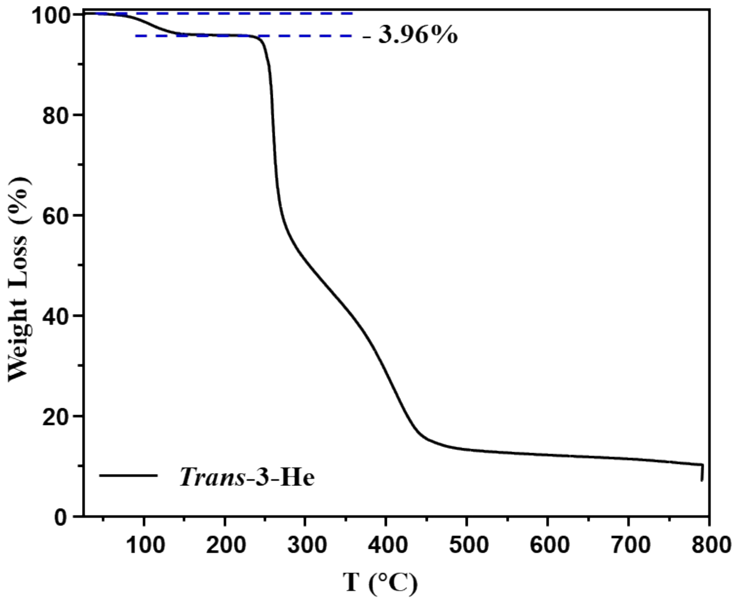

Figure S16. Thermogravimetric analysis of activated **P-TA** after adsorption of *trans*-3-He.

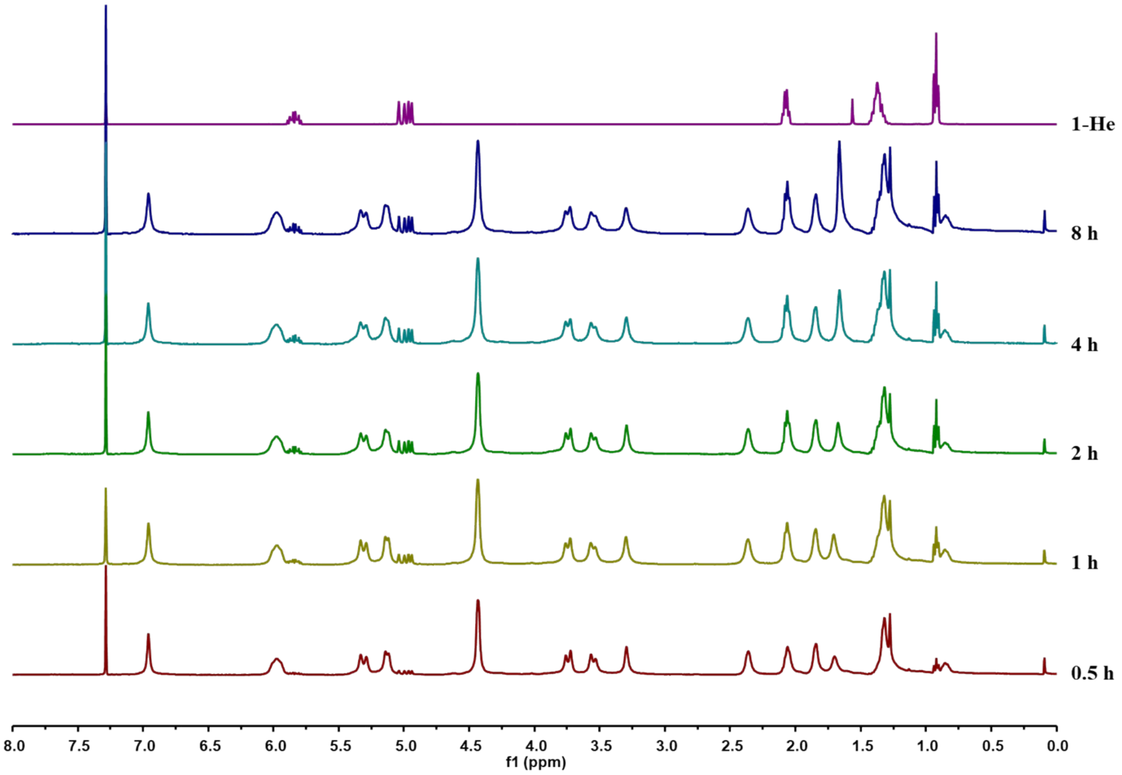

Figure S17. ^{1}H NMR spectra (400 MHz, chloroform-d, 298 K) of activated **P-TA** after adsorption of 1-He over time.

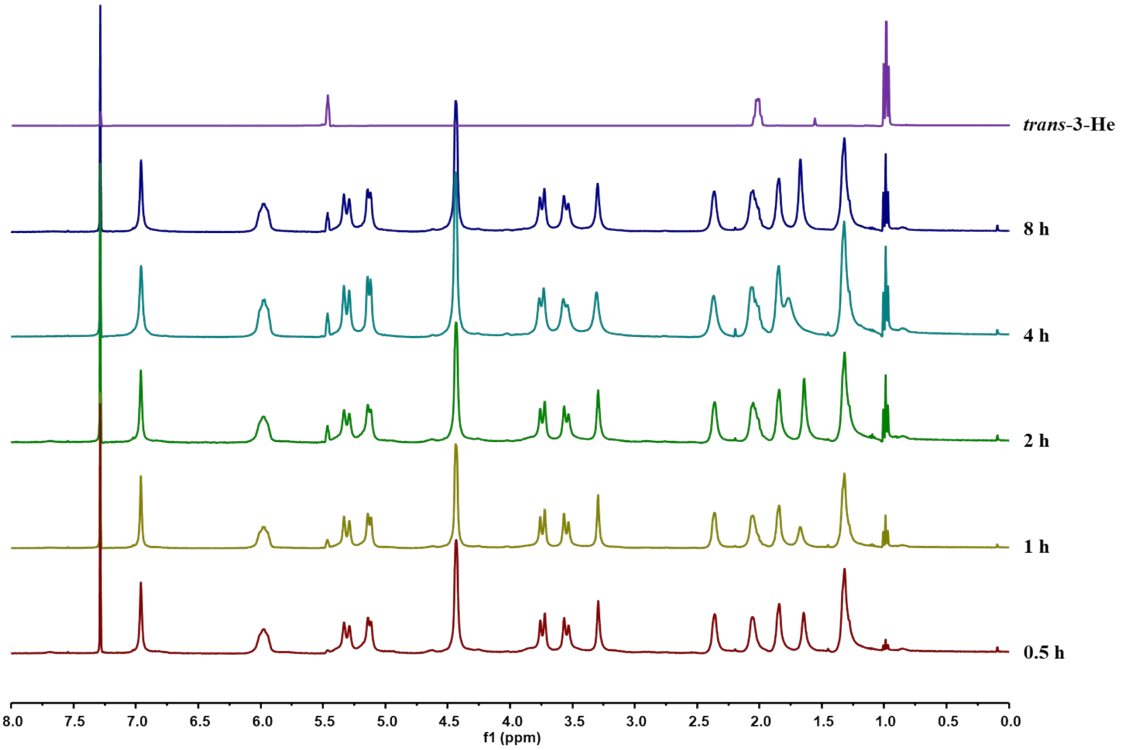

Figure S18. ^{1}H NMR spectra (400 MHz, chloroform-d, 298 K) of activated **P-TA** after adsorption of *trans*-3-He over time.

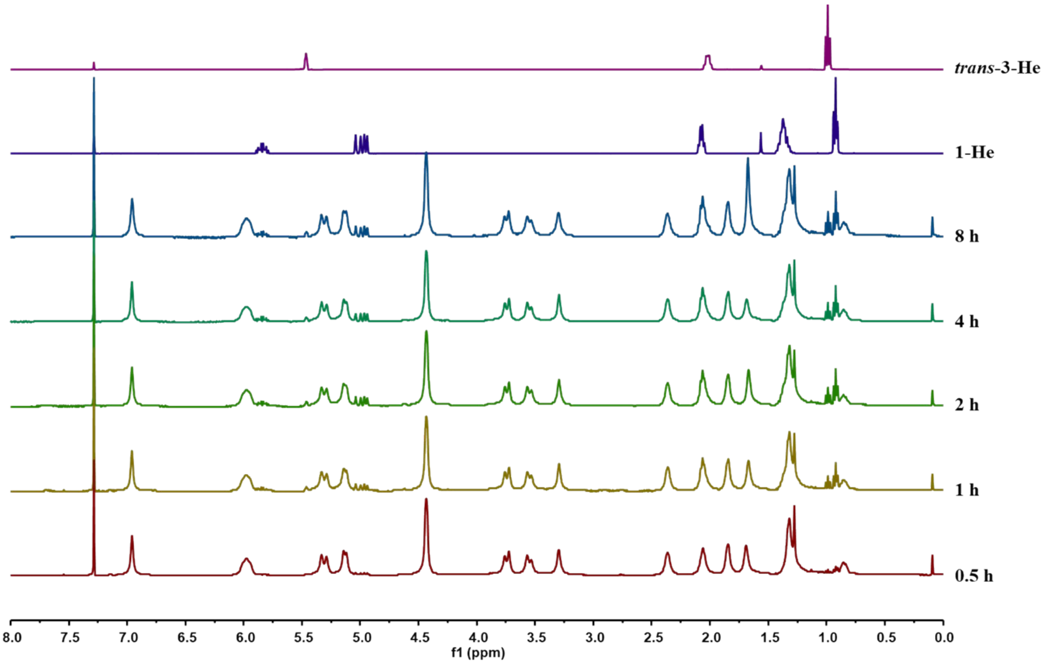

Figure S19. ^{1}H NMR spectra (400 MHz, chloroform-d, 298 K) of activated **P-TA** after adsorption of 1-He/*trans*-3-He mixtures over time.

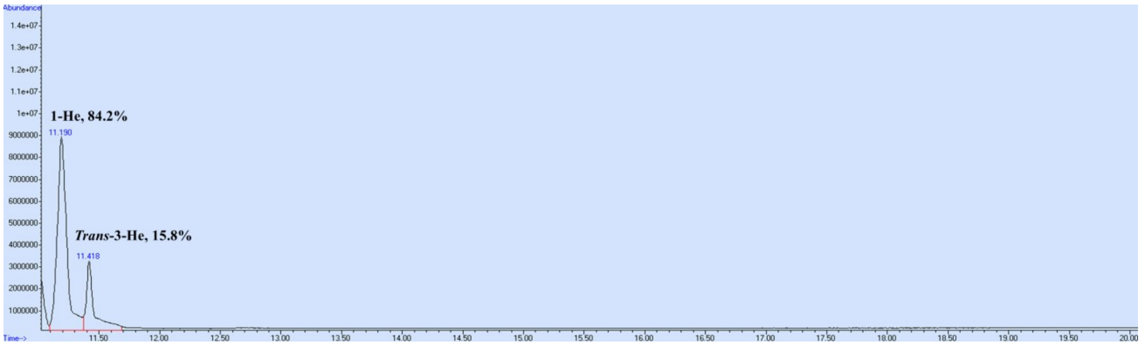

Figure S20. Gas chromatography showing the relative uptake of 1-He and *trans*-3-He by activated **P-TA** from their mixtures for 16h.

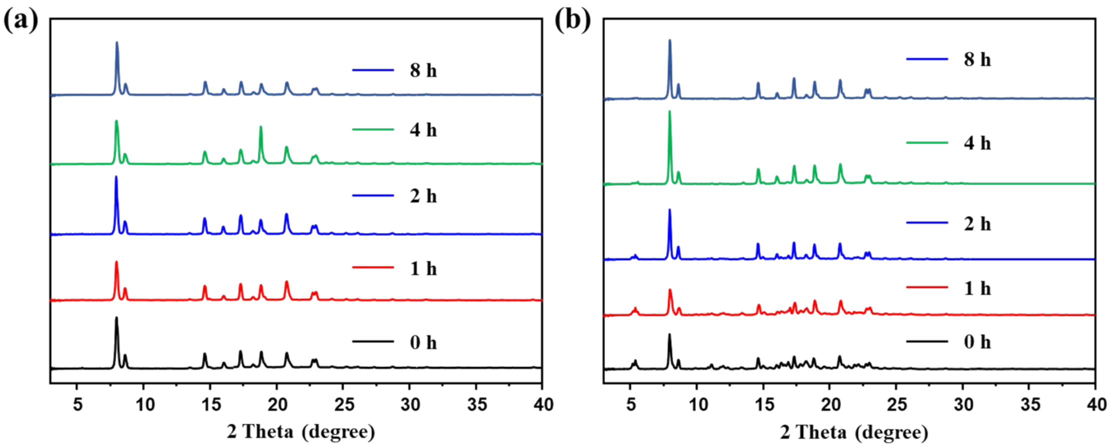

Figure S21. (a) Time-dependent PXRD patterns of **P-TA** loaded with 1-He after exposure to *trans*-3-He vapor under different times. (b) Time-dependent PXRD patterns of **P-TA** loaded with *trans*-3-He after exposure to 1-He vapor under different times.

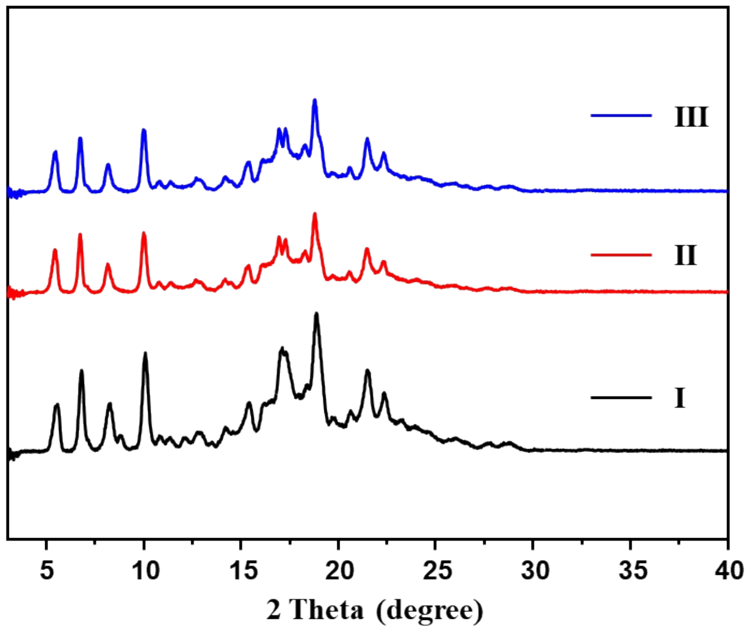

Figure S22. Experimental PXRD patterns of guest-loaded **P-TA** (exposing to 1-He and *trans*-3-He mixtures) after guests were fully removed at 90 °C under vacuum: (I) the first cycle; (II) the third cycle; (III) the fifth cycle.

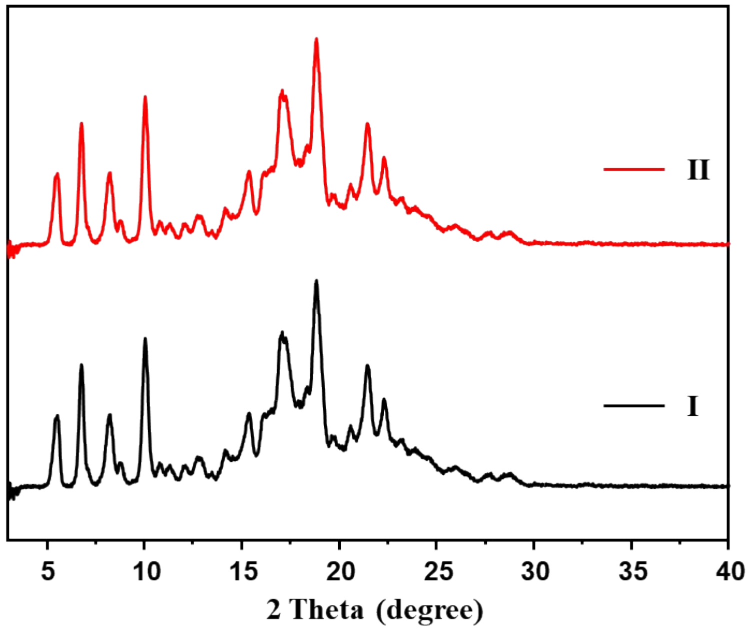

Figure S23. Water stability of: (I) activated **P-TA**; (II) activated **P-TA** soaked in water for 7 days.

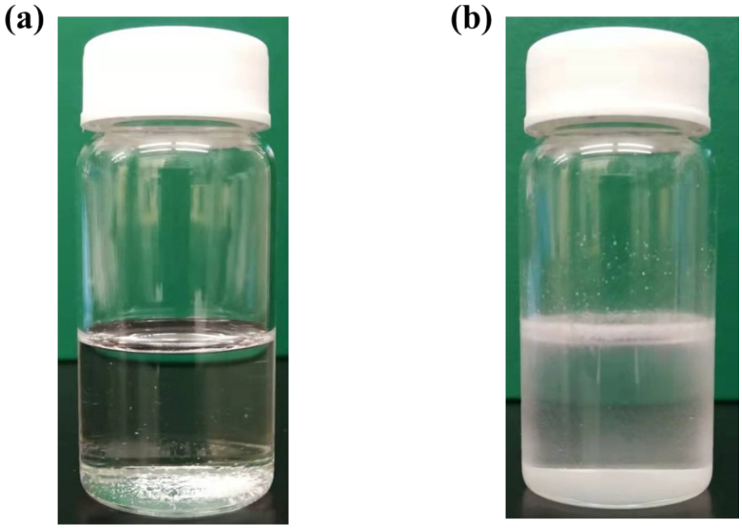

Figure S24. Activated **P-TA** (1mg) in 1-He (10 mL) (a) before and (b) after sonication for 10 min.

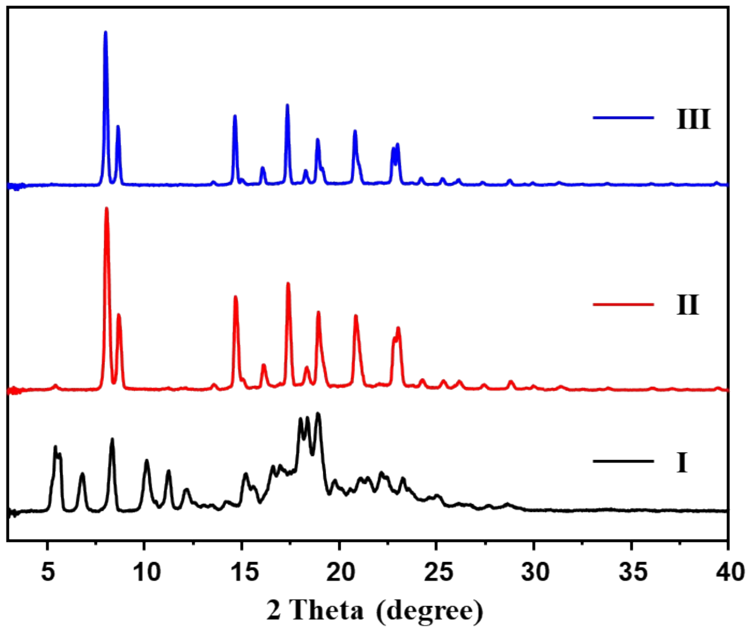

Figure S25. Experimental PXRD patterns of activated **P-TA** after exposure to 1-He/*trans*-3-He mixtures for 16 h: (I) activated **P-TA**; (II) activated **P-TA** in vapor; (III) activated **P-TA** in liquid.

Synthesis of SBA-15. SBA-15 was prepared according to a previously published report.[6]

Synthesis of modified SBA-15. Activated **P-TA** (150 mg) was firstly dissolved in DCM (10 mL), then SBA-15 (150mg) was added into the above solution. The mixture was then sonicated followed by slow evaporation of dichloromethane and washing by DCM (3 times). The as-synthesized **P-TA** loaded SBA-15 was finally dried under vacuum at 120 ℃ overnight.

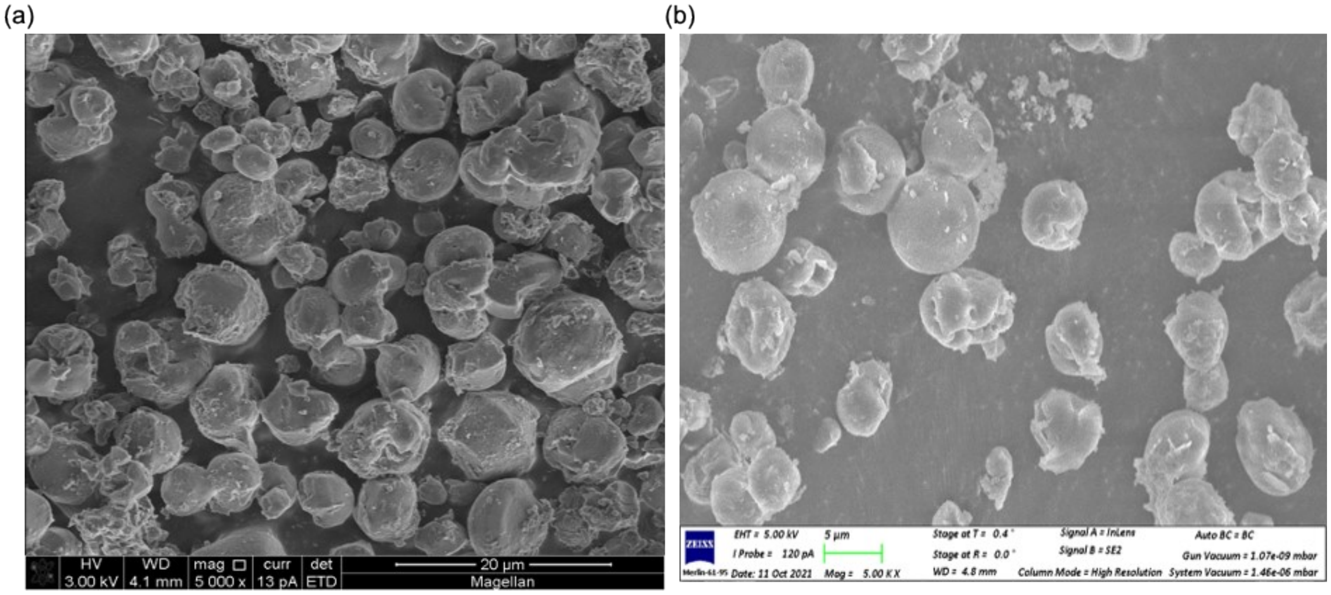

Figure S26. SEM images (a) SBA-15; (b) **P-TA** modified SBA-15 showing no **P-TA** crystallization on the surface.

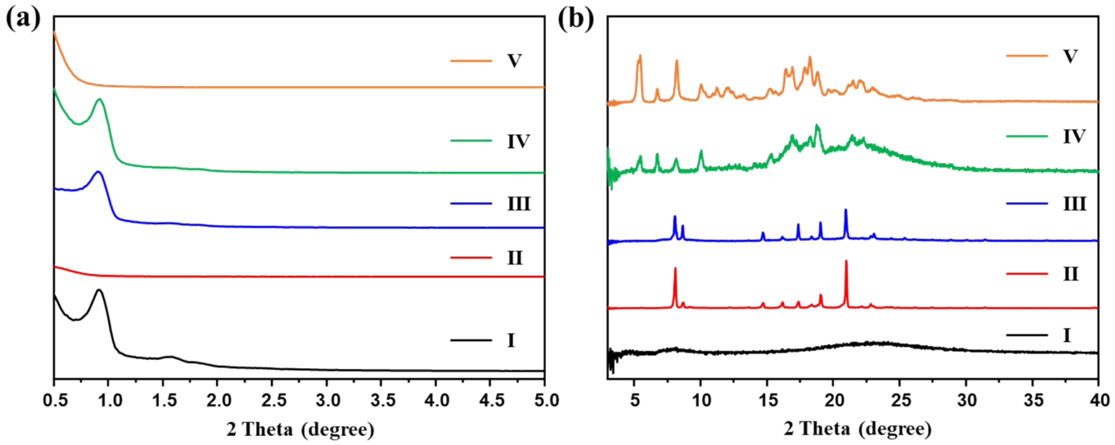

Figure S27. (a) small-angle and (b) broad-angle PXRD patterns: (I) SBA-15; (II) **P-TA** crystals; (III) as-synthesized **P-TA** loaded SBA-15; (IV) modified SBA-15; (V) activated **P-TA.**

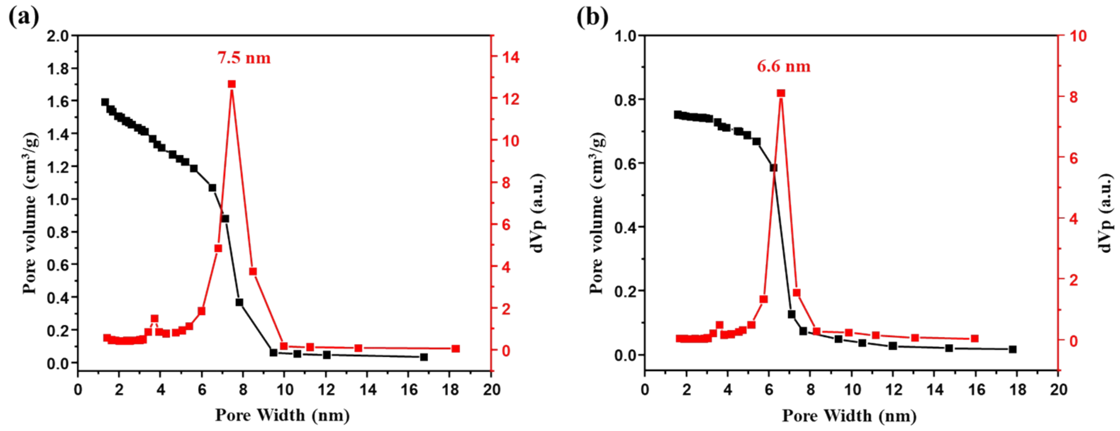

Figure S28. Pore size distributions of (a) SBA-15 and (b) modified SBA-15.

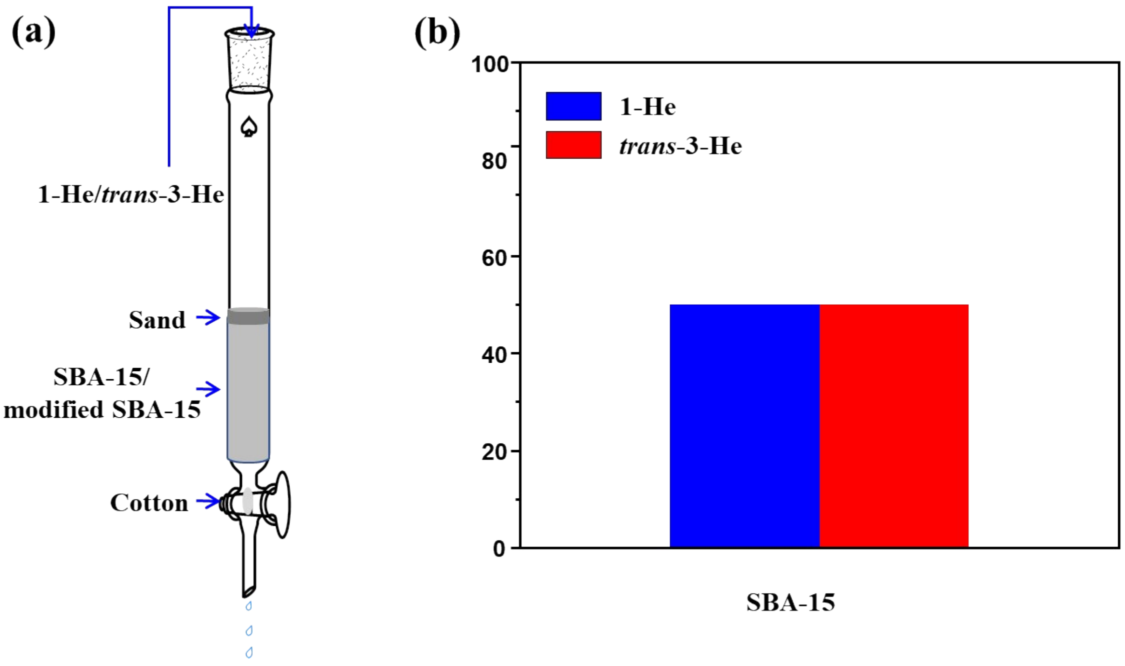

Figure S29. (a) Setup of column chromatography using SBA-15 or modified SBA-15 as the stationary phase. (b) Relative amount of 1-He and *trans*-3-He after the first run with unmodified SBA-15 as the stationary phase.

Table S1. Experimental single crystal X-ray data.

Identification code	**TA**	**P-TA**
Empirical formula	$C_{45}H_{60}N_6 \cdot CH_2Cl_2$[a]	$C_{63}H_{84}N_6O_6 \cdot CH_2Cl_2$[a]
Formula weight	769.91	1106.28
Temperature /K	120.05	120.0
Crystal system	Orthorhombic	Trigonal
Space group	$P\ 2_12_12_1$	$P\ 31$
a /Å	9.0657(2)	20.2894(6)
b /Å	18.4002(4)	20.2894(6)
c /Å	25.5482(5)	13.6577(5)
α /°	90.00	90.00
β /°	90.00	90.00
γ /°	90.00	120.00
Volume /Å^3	4261.71(16)	4869.1(3)
Z	4	3
ρ_{calc} g/cm^3	1.200	1.132
μ /mm^{-1}	0.192	0.151
F(000)	1656	1782
Radiation	MoKα (λ = 0.71073Å)	MoKα (λ = 0.71073Å)
Theta range for data collection/°	2.35 to 27.45	2.32 to 27.09
Index ranges	$-11 \leq h \leq 11$, $-23 \leq k \leq 23$, $-31 \leq l \leq 33$	$-26 \leq h \leq 21$, $-26 \leq k \leq 26$, $-17 \leq l \leq 17$
Reflections collected	39169	63281
Independent reflections	9752 [R_{int} = 0.0368, R_{sigma} = 0.0325]	14887 [R_{int} = 0.0667, R_{sigma} = 0.0533]
Data/restraints/parameters	8444/44/514	11228/117/731
Goodness-of-fit on F^2	1.048	1.052
Final R indexes [I>=2σ (I)][b]	R_1 = 0.0447, wR_2 = 0.1044	R_1 = 0.0699, wR_2 = 0.1820
Final R indexes [all data][b]	R_1 = 0.0572, wR_2 = 0.1147	R_1 = 0.1001, wR_2 = 0.2123
CCDC	2113421	2115290

[a] Formula is given based on single-crystal X-ray data.

[b] $R_1 = \Sigma||F_o| - |F_c||/ \Sigma|F_o|$, $wR_2 = \{ \Sigma[w(F_o^2-F_c^2)^2]/ \Sigma[w(F_o^2)^2]\}^{½}$

Table S2. Experimental single crystal X-ray data.

Identification code	**1-He@P-TA**	***trans*-3-He@P-TA**
Empirical formula	$C_{63}H_{84}N_6O_6 \cdot C_6H_{12}$[a]	$C_{63}H_{84}N_6O_6 \cdot 0.5(C_6H_{12})$[a]
Formula weight	1105.51	1063.43
Temperature /K	120.0	120.0
Crystal system	Trigonal	Trigonal
Space group	*R 3*	*R 3*
a /Å	20.366	20.3338(3)
b /Å	20.366	20.3338(3)
c /Å	13.791	13.7372(4)
α /°	90.00	90.00
β /°	90.00	90.00
γ /°	120.00	120.00
Volume /Å^3	4953.9	4918.9(2)
Z	3	3
ρ_{calc} g/cm^3	1.112	1.077
μ /mm^{-1}	0.553	0.540
F(000)	1800	1728
Radiation	CuKα (λ = 1.54178 Å)	CuKα (λ = 1.54178 Å)
Theta range for data collection/°	4.344 to 65.024	5.97 to 65.10
Index ranges	$-23\leq h \leq 23$, $-23\leq k \leq 23$, $-16 \leq l \leq 16$	$-22\leq h \leq 23$, $-23\leq k \leq 23$, $-16 \leq l \leq 16$
Reflections collected	16121	21516
Independent reflections	3722 [R_{int} = 0.0241, R_{sigma} = 0.0206]	3648 [R_{int} = 0.0215, R_{sigma} = 0.0145]
Data/restraints/parameters	3721/1049/282	3650/908/283
Goodness-of-fit on F^2	1.102	1.074
Final R indexes [I>=2σ (I)][b]	R_1 = 0.0381, wR_2 = 0.1073	R_1 = 0.0708, wR_2 = 0.2055
Final R indexes [all data][b]	R_1 = 0.0381, wR_2 = 0.1073	R_1 = 0.0708, wR_2 = 0.2056
CCDC	2120030	2113573

[a] Formula is given based on single-crystal X-ray data.
[b] $R_1 = \Sigma||F_o| - |F_c||/ \Sigma|F_o|$, $wR_2 = \{ \Sigma[w(F_o^2-F_c^2)^2]/ \Sigma[w(F_o^2)^2]\}^{½}$

Reference

[1] Hua, B.; Ding, Y.; Alimi, L.; Moosa, B.; Zhang, G.; Sessler, J. L.; Khashab, N. M. *Chem. Sci.* **2021**, *12*, 12286 – 12291.

[2] Jie, K.; Liu, M.; Zhou, Y.; Little, M. A.; Bonakala, S.; Chong, S. Y.; Stephenson, A.; Chen, L.; Huang, F.; Cooper, A. I. *J. Am. Chem. Soc.* **2017**, *139*, 2908 – 2911.

[3] SAINT. Bruker AXS. Inc, Madison, Wisconsin, USA, **2014**.

[4] SADABS. G. M. Sheldrick, University of Gottingen, Germany, **2008**.

[5] Sheldrick, G. M. A Short History of SHELX. *Acta Crystallogr.* **2008**, *A64*, 112 – 122.

[6] Chaudhary, V., Sharma, S. *J. Porous Mater.* **2017**, *24*, 741-749.
